# Decreased frontotemporal connectivity in patients with parkinson’s disease experiencing face pareidolia

**DOI:** 10.1038/s41531-021-00237-z

**Published:** 2021-10-07

**Authors:** Yuta Kajiyama, Noriaki Hattori, Tomohito Nakano, Gajanan S. Revankar, Hironori Otomune, Ryota Hashimoto, Etsuro Mori, Manabu Ikeda, Masahito Mihara, Hideki Mochizuki

**Affiliations:** 1 Department of Neurology, Osaka University Graduate School of Medicine, Osaka, Japan; 2 Department of Rehabilitation, Faculty of Medicine, Academic Assembly, University of Toyama, Toyama, Japan; 3 Department of Neurology, Suita Municipal Hospital, Suita, Japan; 4 Department of Pathology of Mental Disease, National Center of Neurology and Psychiatry, Tokyo, Japan; 5 Department of Behavioral Neurology and Neuropsychiatry, United Graduate School of Child Development, Osaka University, Osaka, Japan; 6 Department of Psychiatry, Osaka University Graduate School of Medicine, Osaka, Japan; 7 Department of Neurology, Kawasaki Medical School, Kurashiki, Japan

**Keywords:** Parkinson's disease, Brain, Magnetic resonance imaging

## Abstract

The precise neural underpinnings of face pareidolia in patients with Parkinson’s disease (PD) remain unclear. We aimed to clarify face recognition network abnormalities associated with face pareidolia in such patients. Eighty-three patients with PD and 40 healthy controls were recruited in this study. Patients with PD were classified into pareidolia and nonpareidolia groups. Volumetric analyses revealed no significant differences between the pareidolia (*n* = 39) and nonpareidolia (*n* = 44) patient groups. We further observed decreased functional connectivity among regions of interest in the bilateral frontotemporal lobes in patients with pareidolia. Seed-based analysis using bilateral temporal fusiform cortices as seeds revealed significantly decreased connectivity with the bilateral inferior medial prefrontal cortices in the pareidolia group. Post hoc regression analysis further demonstrated that the severity of face pareidolia was negatively correlated with functional connectivity between the bilateral temporal fusiform and medial prefrontal cortices. Our findings suggest that top-down modulation of the face recognition network is impaired in patients with PD experiencing face pareidolia.

## Introduction

Parkinson’s disease (PD) is a progressive neurodegenerative disorder with an estimated prevalence of approximately 1% among adults over the age of 60 years^1^. In addition to the well-documented motor signs associated with PD, patients often exhibit nonmotor symptoms—either autonomic, cognitive, or neuropsychiatric^2^—even in the early stages of the disease^3^.

Characterized by the visual misperception of an ambiguous stimulus as a face, face pareidolia has a prevalence of as high as 30% among patients with PD: a higher rate than that observed among healthy controls^4^. Several studies have reported that patients with idiopathic rapid eye movement sleep behavior disorder (iRBD) experience pareidolia more frequently than healthy individuals, and that there may be an association between pareidolia and cognitive decline^5^. In a study involving patients with dementia with Lewy bodies (DLB), face pareidolia was associated with the severity of visual hallucinations and visuospatial deficits^6^. These studies suggest a possible association between face pareidolia and Lewy body pathology; however, these studies were conducted under heterogeneous clinical conditions, and the precise mechanism of face pareidolia remains unclear.

The neural system for face recognition has been well documented and consists of several brain regions in the occipitotemporal cortex: the fusiform gyrus (fusiform face area: FFA), the inferior occipital cortex (occipital face area), and the posterior superior temporal sulcus. Among these areas, the FFA is considered crucial for face perception and identification^7–10^. In addition to these networks, which are mainly involved in “bottom-up” sensory integration for face perception, recent studies have suggested the importance of “top-down” modulations from the frontal region for face perception^11–13^. Previous studies that investigated the neural mechanisms of face pareidolia have revealed that the abovementioned face recognition network is involved in the pareidolic reaction in healthy participants. Using the illusory face perception paradigm with pure-noise visual stimuli, Liu et al. described a distributed network including the frontal, parietal, temporal, and occipital cortices in those with face pareidolia^14^. Other studies with similar paradigms have also suggested an association between FFA activation and face pareidolia;^15,16^ however, substantial interindividual variability has also been suggested^17^. These findings imply a possible association between dysfunction of the face recognition network and pareidolia in PD.

Recently, noninvasive imaging techniques such as resting-state functional magnetic resonance imaging (rsfMRI) have enabled us to investigate the status of brain networks in patients with PD. Previous studies have suggested the early alteration of wide-spread brain networks in PD^18,19^, as well as its association with motor and nonmotor symptoms^20–22^. Given that brain network alterations can occur in the early stages of PD prior to the development of cognitive impairment, alterations in the face recognition network may be associated with face pareidolia in patients with PD.

It is possible that both “top-down” modulation and “bottom-up” sensory integration streams to the FFA play a vital role in the development of face pareidolia in patients with PD. Informed by our previous electroencephalogram findings that suggest the involvement of “top-down” attentional modulation from the frontal cortices^23^, we hypothesized that dysfunction of “top-down” modulation associated with the FFA is involved in face pareidolia. Using rsfMRI, the present study aimed to uncover the network abnormalities associated with pareidolia in patients with early-stage PD without dementia or hallucinations.

## Results

### Clinical characteristics

Among the 97 selected patients with PD, 14 were excluded from further analysis due to poor quality of the imaging data (e.g., excessive head movement). No participants in the healthy control (HC) group exhibited excessive head motion. Ultimately, 83 patients with PD (34 men and 48 women; age: 67.2 ± 9.8 years; Hoehn and Yahr (HY) stage:^24^ 2.6 ± 0.9); and 40 HCs (18 men and 22 women; age: 66.0 ± 3.7 years) were included in the analysis. Based on the Noise Pareidolia Test (NPT) screening, 39 patients with PD (47.6%) were classified into the PD with pareidolia (PD-p + ) group, while 44 patients (52.4%) were classified into the PD without pareidolia (PD-p-) group. The only characteristic that differed significantly between the PD groups was the Mini-Mental State Examination (MMSE) score^25^ (27.0 ± 2.2 vs. 28.8 ± 1.7, *p* < 0.01) (Table 1).

**Table 1:** Demographic characteristics and variables for patients with Parkinson’s disease with and without pareidolia (*n* = 83).

	PD with pareidolia (*n* = 39)	PD without pareidolia (*n* = 44)	Healthy control (*n* = 40)
Age (years ± SD)	68.3 ± 10.2	66.2 ± 9.2	66.0 ± 3.7
Gender (men: women)	16:23	19:25	18:22
Symptom laterality (more affected side; right: left)	24:15	21:23	–
Hoehn and Yahr Stage (±SD)	2.6 ± 0.9	2.6 ± 1.0	–
Duration of motor symptoms (years ± SD)	4.7 ± 4.3	5.5 ± 6.8	–
NPT score			
Pareidolia (±SD)	6.76 ± 7.32	0	–
Miss (±SD)	0.50 ± 0.85	0.22 ± 0.51	–
Hallucination and psychosis (MDS-UPDRS 1.2)			
Score 0	33	40	–
Score 1	3	4	
Score 2	3	0	
≧Score 3	0	0	
MMSE ( ± SD)	27.0 ± 2.2 *	28.8 ± 1.7*	–
JLO ( ± SD)	10 ± 2.9	11.1 ± 3.1	–
MDS-UPDRS			
Part I ( ± SD)	10.3 ± 4.3	9.5 ± 5.3	–
Part II ( ± SD)	12.9 ± 6.8	14.2 ± 8.0	
Part III ( ± SD)	31.7 ± 14.3	27.3 ± 12.5	
FAB ( ± SD)	15.1 ± 2.0	15.4 ± 2.1	–

*PD* Parkinson’s disease, *SD* standard deviation, *NPT* Noise Pareidolia Test, *MDS-UPDRS* Movement Disorder Society-sponsored revision of the Unified Parkinson’s Disease Rating Scale, *MMSE* Mini-Mental State Examination, *JLO* Benton Judgement of Line Orientation test, *FAB* Frontal Assessment Battery.

*p < 0.05.

Scores are presented as means ± SD.

### Gray matter volume analysis

No significant differences in gray matter volume were observed between the PD-p+ and PD-p− groups. Compared with the HC group, the PD-p+ group exhibited reduced gray matter volume in the left temporal pole, left central operculum, and brainstem, while the PD-p− group exhibited reduced gray matter volume in the left central operculum and brainstem.

### Functional connectivity analysis

**Fig. 1:**
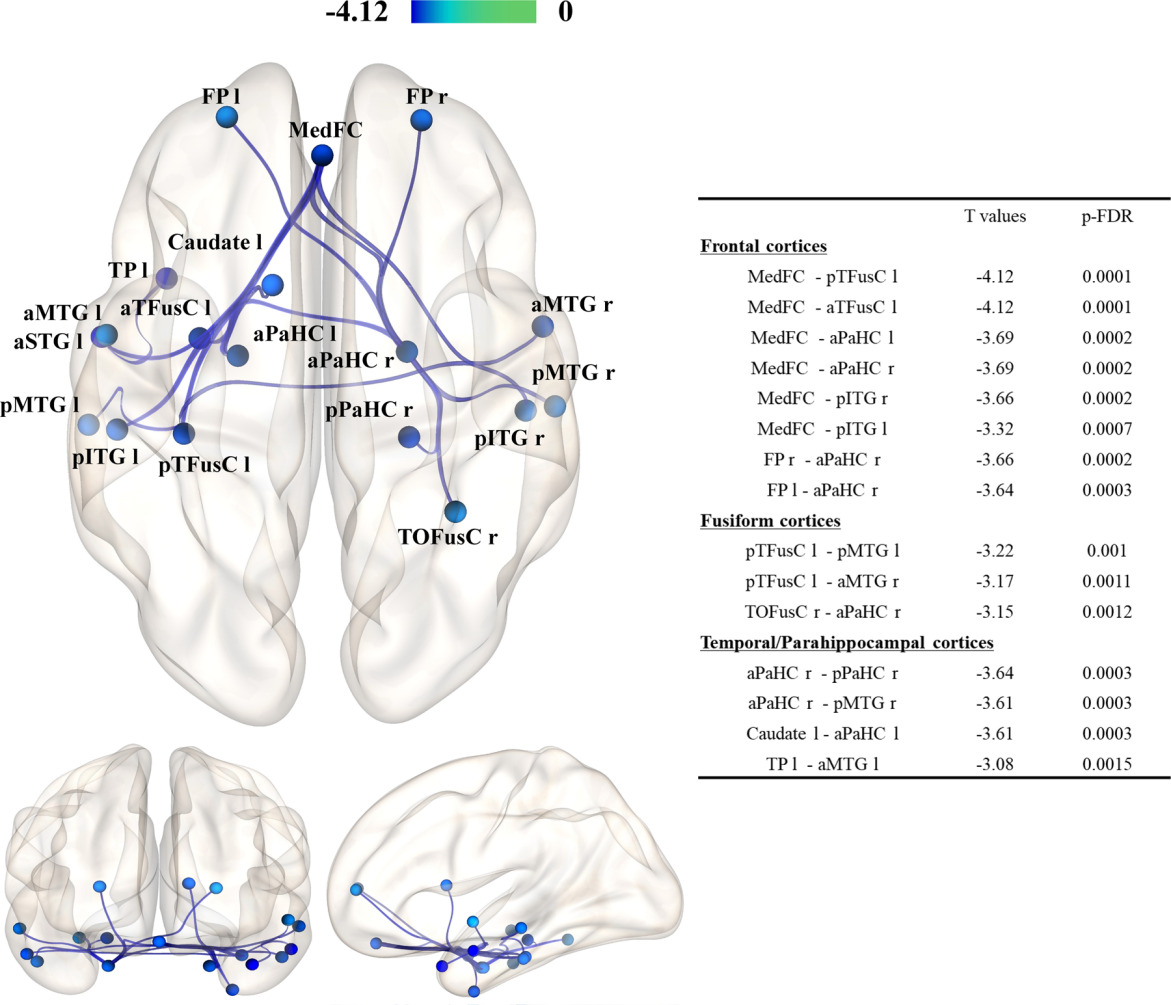
Decreased functional connectivity in patients with PD with pareidolia compared to those without pareidolia. The decrease in connectivity was statistically significant between PD-p + and PD-p- groups (PD-p + < PD-p−: controlled for age, MMSE). The T values of the negative difference in functional connectivity are shown as a colored bar. Patients in the pareidolia group exhibited decreased functional connectivity between the medial prefrontal cortices and the bilateral temporal lobe (including the left temporal fusiform cortices, bilateral parahippocampal cortices, and right inferior temporal gyri) when compared with patients in the nonpareidolia group. Abbreviations: PD, Parkinson’s disease; MMSE Mini-Mental State Examination, r right, l left, FP frontal pole, MedFC frontal medial cortex, a/pPaHC parahippocampal gyrus anterior/posterior division, pITG inferior temporal gyrus, posterior division, a/pMTG middle temporal gyrus, anterior/posterior division, a/pTFusC temporal fusiform cortex anterior/posterior division, TOFusC temporal occipital fusiform cortex, TP temporal pole, aSTG superior temporal gyrus anterior.

In the region of interest (ROI)-based analysis, both the PD-p+ and PD-p− groups exhibited substantial differences in functional connectivity throughout the brain when compared to the HC group. The comparison between the two PD groups revealed that the PD-p+ group exhibited decreased functional connectivity in the networks of multiple regions in the frontal and temporal lobes, relative to that observed in the PD-p− group. The most significant differences in functional connectivity were observed in the network between the medial frontal cortices (MedFC) and the left temporal fusiform cortex (TFusC) (Fig. 1).

**Fig. 2:**
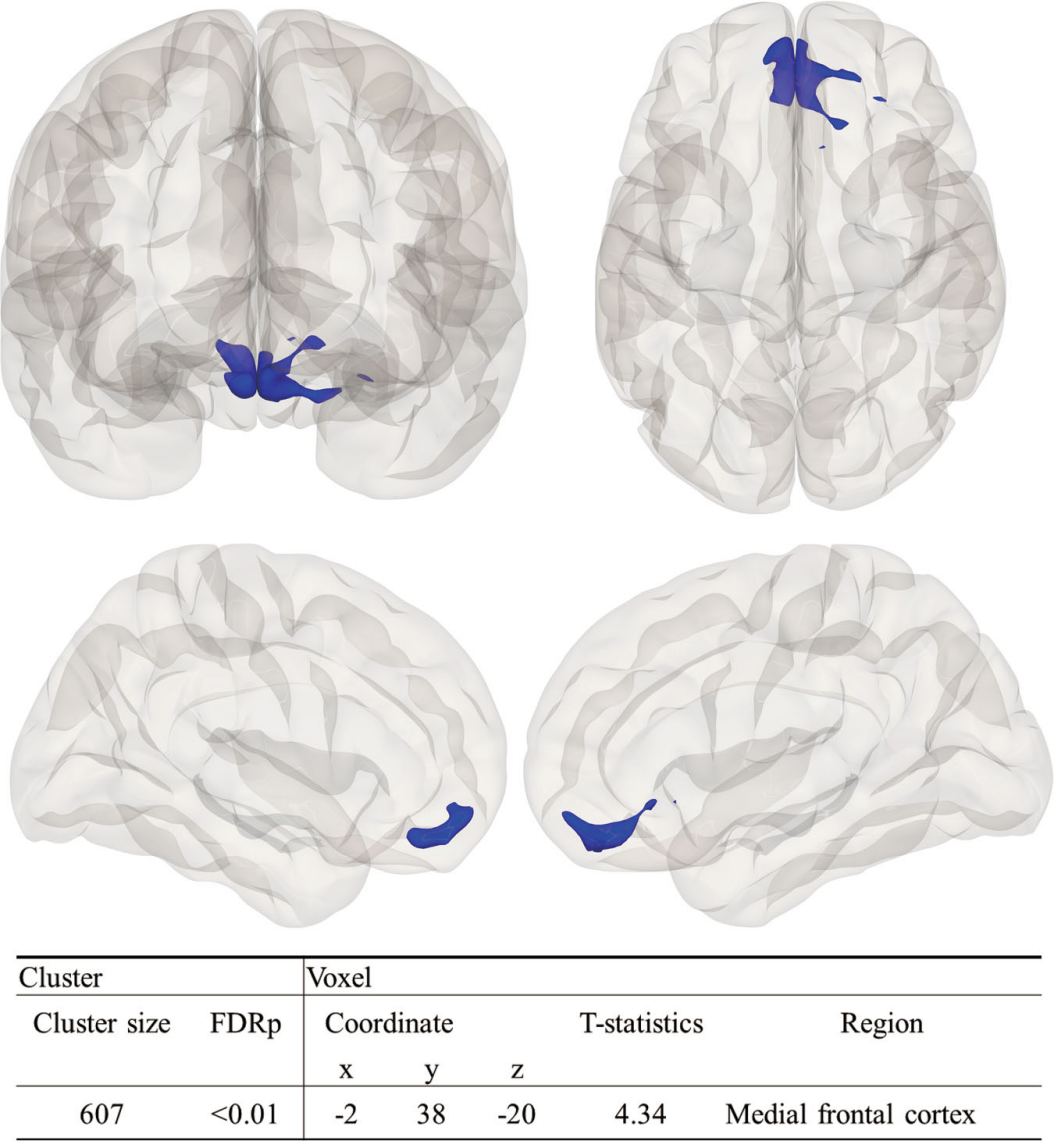
Cortical areas showing decreased connectivity with the bilateral temporal fusiform cortices in pareidolia. Spatial maps for seed-based connectivity analyses with the bilateral temporal fusiform cortices as the seed regions (PD-p + < PD-p−: controlled for age, MMSE). The inferior medial prefrontal cortices exhibited significantly decreased connectivity with the bilateral temporal fusiform cortices. FDR-p false discovery rate-corrected *p* value.

**Fig. 3:**
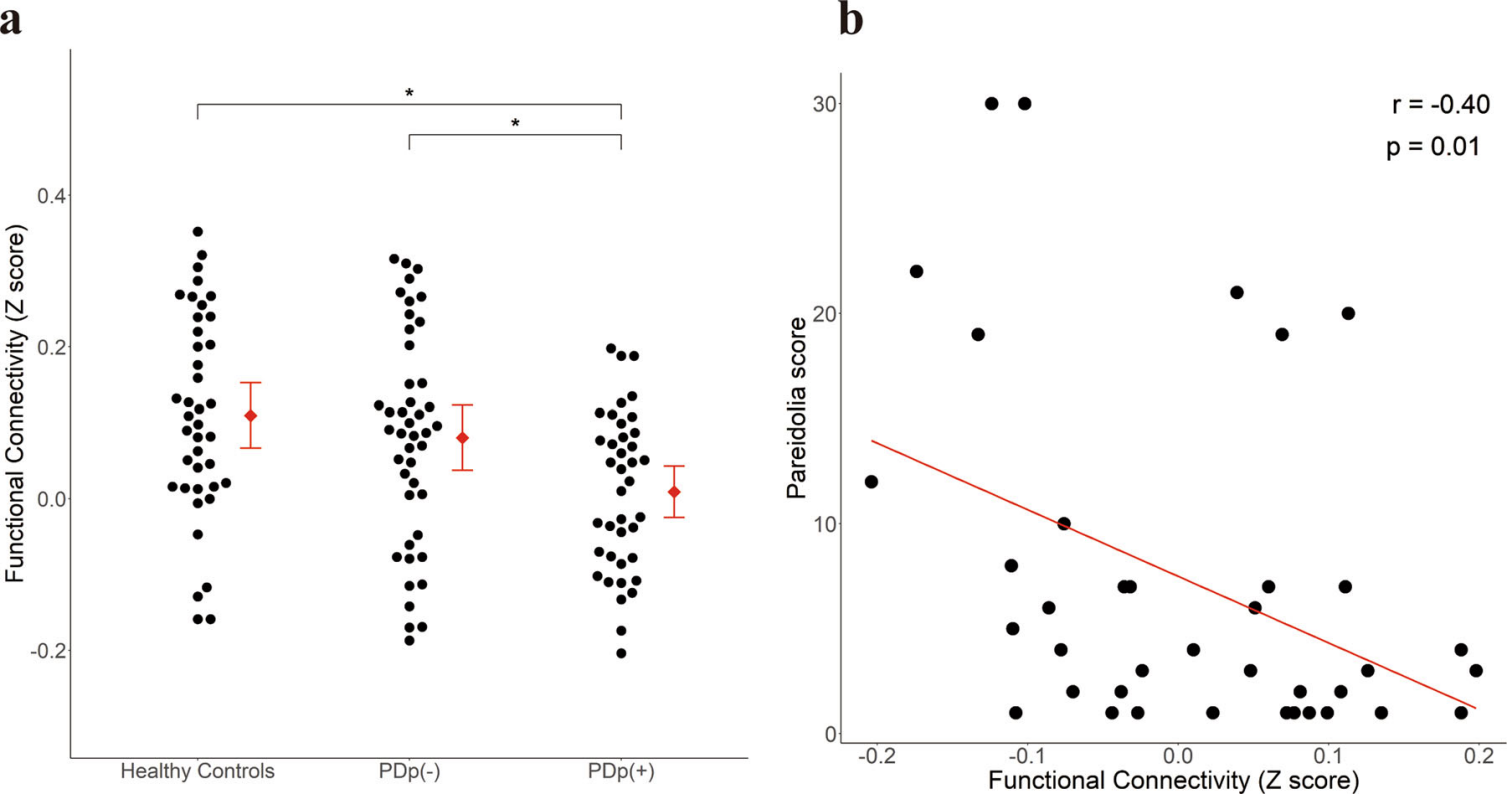
Groupwise comparison of front-temporal networks and its correlation with the pareidolia score. FC shows *Z*-scores of the correlation coefficient between the medial frontal cortex and the bilateral fusiform cortices. **a** (left): Dot-plot shows comparison of FC among the three groups. The mean of each group with 95% confidence intervals are shown as red rhombi with error bar. * FDR-p < 0.05. (ANOVA with FDR adjusted post hoc Tukey–Kramer test). **b** (right): Correlation between frontotemporal connectivity and pareidolia score. Scatter plot shows Pearson’s correlation between FC and pareidolia scores in patients with pareidolia. FC functional connectivity, ANOVA analysis of variance, FDR false discovery rate.

Consistent with the ROI-to-ROI (ROI-based) analysis, in the seed-based analysis using the bilateral TFusC as a seed, the bilateral inferior MedFC exhibited significantly decreased connectivity in the PD-p+ group, when compared with that in the PD-p− group (Fig. 2). Subsequently, we performed a group comparison of *Z*-scores for functional connectivity values (Fisher’s transformation of the correlation coefficient) between the TFusC and the bilateral inferior MedFC. The mean Z-scores [95% confidence interval (CI)] of each group were as follows, PD-p + : 0.009 [−0.025 to 0.043], PD-p-: 0.080 [0.037 to 0.123], and HC group: 0.110 [0.067 to 0.153]. Groupwise comparison using analysis of variance (ANOVA) revealed a significant difference among the three groups (F(2120) = 6.42; *p* = 0.002). Post hoc analysis showed that the PD-p + group exhibited significantly decreased connectivity compared with those of the PD-p− and HC groups, while there was no difference in connectivity between the PD-p− and HC groups (PD-p + vs. PD-p- group: FDR-*p* < 0.05; 95% CI [−0.138 to −0.004], effect size, *d* = −0.57; PD-p + vs. HC group: FDR-*p* < 0.05; 95%CI [−0.169 to −0.031], effect size, *d* = −0.84: PD-p- vs. HC group: FDR-*p* = 0.84; 95%CI [−0.096 to 0.037], effect size, *d* = −0.21) (Fig. 3a). Additional post hoc analysis showed no significant effect of symptom lateralization on functional connectivity between the TFusC and the MedFC (*t*(80.435) = 1.59, 95% CI [−0.012 to 0.108], *p* = 0.11). Functional connectivity *Z*-scores were also significantly negatively correlated with NPT scores in the PD-p+ group (*r* = −0.40, *p* = 0.01) (Fig. 3b).

**Fig. 4:**
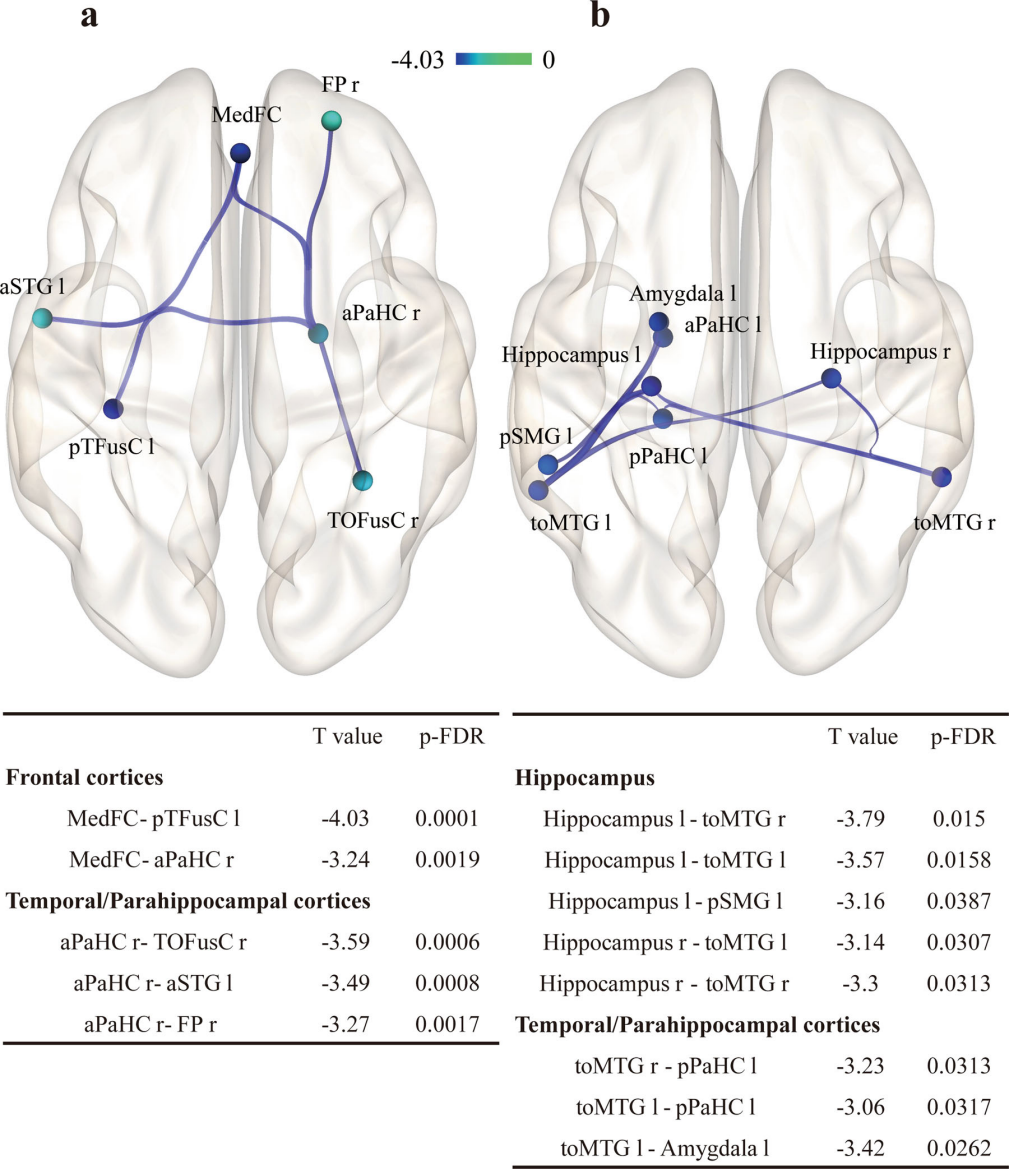
Functional networks showing pareidolia score- and MMSE score- dependent alteration. **a** (left): Regression analysis of functional connectivity revealing pareidolia score-dependent decrement. Functional connectivity between the MedFC and pTFusC was negatively correlated with the pareidolia score. **b** (right): Regression analysis of functional connectivity revealing the MMSE score-dependent decrement. Functional connectivity between the bilateral hippocampus and middle temporal gyrus was negatively correlated with MMSE score. *T* values of the negative difference in functional connectivity are shown as a colored bar. The statistical significance was set to an FDR-p < 0.05. MMSE Mini-Mental State Examination, r right, l left, MedFC medial frontal cortices, pTFusC temporal fusiform cortex posterior division, TOFusC temporal occipital fusiform cortex, aSTG superior temporal gyrus anterior, FP frontal pole, pSMG supramarginal gyrus posterior division, toMTG middle temporal gyrus temporooccipital part, aPaHC right parahippocampal gyrus anterior division, pPaHC parahippocampal gyrus posterior division.

Regression analysis using functional connectivity values for the pareidolia score revealed a significant negative correlation with functional connectivity in the network including the MedFC, left TFusC, right parahippocampal gyrus, anterior division, and right temporal occipital fusiform cortex (Fig. 4a). In contrast, regression analysis for MMSE scores revealed a significant negative correlation with connectivity in the different networks around the bilateral hippocampus and middle temporal gyrus (Fig. 4b).

## Discussion

The present study aimed to investigate network abnormalities associated with pareidolia in patients with early-stage PD, prior to the development of dementia or hallucinations. Our findings revealed decreased connectivity between the medial prefrontal cortices and the medial temporal area, including the FFA, in patients with PD experiencing pareidolia. Moreover, the severity of face pareidolia was negatively correlated with functional connectivity between the bilateral temporal fusiform and medial prefrontal cortices.

Although the “bottom-up” information stream to the FFA is emphasized in the classical model of face recognition, recent studies have revealed the importance of “top-down” modulations from frontal regions to the FFA^11,12,23^. A previous study revealed that when humans determine whether the visual objects are faces^26^, their attention focuses on either a face or the scenery in front of them, which alters the neural activity of the FFA and parahippocampal place area, respectively^27^, as well as the preceding neural responses in the medial prefrontal cortices. Zhang et al. also demonstrated an association between activation of the medial prefrontal cortices and pareidolic reactions in pure-noise images during face detection tasks^28^. Interestingly, we observed significantly decreased connectivity between the medial frontal region and the fusiform cortices in the PD-p+ group but not in the HC or PD-p− group. We also identified a significant negative correlation between this functional connectivity and pareidolic reactions. These findings suggest the importance of regulatory control from the medial prefrontal region to the FFA in the pathophysiology of face pareidolia.

The idea that loss of attentional control is associated with face pareidolia is consistent with clinical observations that face pareidolia and visual hallucinations are associated with global cognitive decline, which results in attention deficits^29,30^. Previous studies have highlighted the potential involvement of visual attention-related functional networks including the default mode network, ventral attention network, and dorsal attention network in visual misperception^31,32^. These findings suggest that both the functional connection between the medial frontal region and the fusiform cortices and the involvement of wide-spread global networks are important in the pathophysiology of face pareidolia. However, our findings support the notion that disrupted connectivity between the medial prefrontal cortices and the FFA can specifically lead to pareidolia in patients with PD. Indeed, our study excluded patients with hallucinations, and our post hoc regression analysis revealed that distinct networks were associated with pareidolia and cognitive status.

Previous studies have also emphasized the involvement of classical “bottom-up” visual streaming from the occipital cortex in face pareidolia. A positron emission tomography study reported that occipital hypoperfusion was associated with pareidolia^29^. Another rsfMRI study reported a visual hallucination-associated reduction in connectivity between the occipital and cortical-striatal regions in patients with PD^33^. Neuropsychological studies have also revealed impaired visuoperceptual functions, including line orientation in PD populations^34^, and longitudinal structural MRI data have suggested the involvement of the occipital, parietal, and temporal areas in visuoperceptual dysfunctions in patients with PD^35^. However, our study found no significant difference in functional connectivity between the occipital regions and FFA, and both groups exhibited comparable visual perceptual ability as assessed using the JLO. Moreover, our voxel-based morphometry findings indicated that gray matter volumes were comparable between the PD-p+ and PD-p− groups. Taken together, these results indicate that both “top-down” and “bottom-up” processing streams may be critical for face pareidolia. Therefore, the pathophysiology of pareidolia in patients with PD may be heterogeneous.

Our results showed no significant effect of symptom lateralization on functional network alteration. This might suggest the left dominant involvement of this network on the pathophysiology of face pareidolia. Considering the hemispheric lateralization in the face recognition system, our ROI-based analysis revealed left-dominant decreased connectivity between the MedFC and the TFusC, although previous clinical observations, as well as neuropsychological and neuroimaging studies have emphasized the dominance of the right hemisphere in the face recognition system^36^. Recent reports have suggested functional differences in face perception between the two hemispheres. Meng et al. reported that the left FFA is involved in detecting “low-level” face resemblance, whereas the right FFA is involved in the more precise judgment of faces^37^. Additionally, a recent functional imaging study using an illusory face paradigm in healthy participants revealed that the right FFA has high selectivity for faces, but that the left FFA also responds to nonface objects, suggesting low-selectivity for faces but nonspecific object reactivity^14,38^. Considering that pareidolic phenomena are not specific to faces and may result in the illusory misperception of other types of objects in patients with PD^6,29^, frontal dysregulation to the left FFA is likely involved in the pareidolic reactions experienced by patients with PD.

There were several limitations to this study. First, although we excluded patients with PD experiencing dementia and the MMSE scores in the PD-p+ and PD-p− groups were within the normal range, there was a significant difference in MMSE score between the groups. In this study, to control for the effect of cognitive deficits, we included the MMSE score into the model as a possible confounding factor. As stated above, our post hoc regression analysis revealed an independent functional network associated with severe pareidolia and cognitive status. However, we cannot exclude the possible influence of subcortical-frontal dysfunctions, which is usually underestimated by MMSE. Second, we only investigated resting-state functional connectivity, which did not allow us to analyze brain activity during the emergence of pareidolia. Pareidolia is a form of visual misperception that occurs in a visual stimulus-dependent manner^6,29^. Therefore, it is possible that our findings did not reflect neural networks that are directly correlated with the pareidolic reaction. It should be noted that our findings revealed a significant correlation between the frontotemporal network and face pareidolia; however, this correlation does not necessarily imply causality. Studies using activation paradigms with sufficient spatial and temporal resolution are required to investigate specific network dysfunctions that can cause pareidolia. Third, as our study included only patients without dementia or hallucinations, the underlying pathophysiology of pareidolia may be different from that in patients with cognitive deficits or hallucinations. Further studies that include patients with a broader range of disease severity or longitudinal data will help to clarify this point. Fourth, based on our hypothesis, we only analyzed the cortical network in this study. However, not only lateral geniculate to cortical pathways, but the retinotectal pathway may also be involved in face recognition^39^, and it has been shown that in PD patients, there is loss of dopaminergic retinal cells and impaired contrast sensitivity in PD^40^. Therefore, it is possible that these subcortical structures may play some roles in face pareidolia in PD. Finally, we conducted this study with patients in the “ON” state for dopaminergic medications. Although there was no significant difference in levodopa equivalent doses between the PD-p+ and PD-p− groups in our study, we could not fully exclude possible drug effects on either pareidolia or functional connectivity.

In conclusion, our findings emphasized the importance of alterations in the frontotemporal network in face pareidolia among patients with PD using rsfMRI. In accordance with the findings of experimental studies conducted with healthy participants^14,28,37,38^, our findings also suggest the possible involvement of “top-down” modulation in the pathophysiology of face pareidolia in PD. Although further replication studies are needed, our findings provide insight into the pathophysiology of frequent visual misperception in patients with PD. Future studies focusing on the frontotemporal network may help confirm whether these findings indicate a common underlying pathophysiology of face pareidolia in patients with Lewy body pathology, including PD, DLB, or iRBD. Our findings may also help identify the role of the visuoperceptual networks in the development of visual hallucinations in patients with Lewy body pathology, which may in turn lead to the identification of a potential target for neuromodulative interventions like deep brain stimulation.

## Methods

### Participants

This study was conducted as part of a longitudinal prospective cohort and registry study of PD at Osaka University that registered all patients with PD who were admitted to our hospital. From the patients registered between September 2014 and November 2016, we selected those who complied with the following selection criteria: (1) aged between 40 and 85 years, (2) diagnosed with PD in accordance with the United Kingdom Parkinson’s Disease Society Brain Bank criteria^41^, (3) HY stage from 1 to 3, and (4) no clinical symptoms of dementia. Exclusion criteria were as follows: (1) a history of other neurological, psychiatric, or severe ocular disease; (2) the presence of subjective hallucinations based on interviews with the patient and caregiver; (3) current use of antipsychotic medications; and (4) a score of less than 24 on MMSE. According to these criteria, 97 patients with PD were selected from among 213 patients who were registered during this period. In addition, we selected 40 age- and sex-matched healthy individuals without any history of neurological or psychiatric diseases from another cohort study^42^, as HCs for rsfMRI connectivity analysis.

### Ethics

This study was approved by the Osaka University Hospital ethics review committee and the Osaka University Research Ethics Committee. Written informed consent was obtained in accordance with the Declaration of Helsinki, and a written agreement was obtained from all participants.

### General/baseline evaluations

Clinical symptoms of PD were assessed using the Movement Disorder Society‐sponsored revision of the Unified Parkinson’s Disease Rating Scale (MDS‐UPDRS) parts I to III and the HY stage^43^, while the patients were in the “ON” state. Cognitive function was assessed using the MMSE, Frontal Assessment Battery (FAB)^44^, the Japanese version of the Montreal Cognitive Assessment (MoCA)^45^, and the short form of the Benton Judgement of Line Orientation Test (JLO), form H^46,47^. Furthermore, we assessed symptom laterality. Dopaminergic medications for PD were converted to levodopa equivalent daily doses^48^.

### Evaluation for pareidolia

To evaluate hallucinatory phenomena in the patient group, we used the noise pareidolia test (NPT), which is designed to evoke and measure pareidolia^49^. In the NPT group, participants were required to determine whether a face was present in each stimulus. The perception of a face in nonface stimulus was considered a pareidolic reaction, with the total number of pareidolic reactions was recorded as the pareidolia score. When a participant did not perceive a face in a face-containing stimulus, the result was considered a “miss”. Those who experienced at least one pareidolic response were included in the PD-p+ group, while the rest were included in the PD-p− group.

### Acquisition and processing of MR images

All images were acquired using a 3-T MR scanner (GE Healthcare, Milwaukee, WI, USA). High-resolution anatomical scans were obtained using a sagittal three-dimensional (3D) fast-spoiled gradient-recalled echo-pulse sequence, and gradient-echo echo-planar imaging was used for rsfMRI. The parameters were the same as those in our former study^50^.

### Quality check for MRI scans

We used a foam pillow to avoid excessive head motion. Participants with any artifacts in their MR scans or with excessive head movement creating more than 1 mm of displacement or 2.5° of rotation in any direction during rsfMRI were excluded from further analysis.

### Imaging acquisition and preprocessing

Gray matter volume analysis was performed with a voxel-based morphometry technique using Statistical Parametric Mapping 12 (SPM12; http://www.fil.ion.ucl.ac.uk/spm/) on Matlab R2018a (MathWorks, Natick, MA, USA), as follows: segmentation into cerebrospinal fluid, gray matter, and white matter based on tissue probability maps; spatial normalization of gray matter segments to a gray matter template in the Montreal Neurological Institute space using diffeomorphic anatomical registration through exponentiated lie algebra techniques; and a 3D Gaussian filter of 8 mm full-width at half-maximum for smoothing of images^51,52^.

Functional connectivity data were preprocessed using the CONN-fMRI functional connectivity toolbox v17 (http://www.nitrc.org/projects/conn)^53^. All T1-weighted structural MR images and rsfMRI data were preprocessed as follows: spatial realignment; slice timing correction; normalization using transformation parameters derived from a high-resolution anatomical scan; and smoothing using a 3D Gaussian filter of 8 mm full-width at half-maximum. All resting-state time series were de-noised and band-pass filtered (0.009–0.08 Hz) for blood-oxygen-level-dependent signals.

### Statistical analysis and analysis of MR images

The Mann–Whitney *U* test and Chi-squared test were used for group comparisons of clinical data, while analyses of covariance including possible confounders were used to analyze brain images. The analyses were performed using R version 3.6 (https://www.r-project.org/), and *p* values < 0.05 (two-sided) were considered statistically significant.

To analyze gray matter volume in the PD-p + , PD-p−, and HC groups, we performed analyses of covariance using age and total brain volume as covariates of no interest to minimize the effects of aging and the brain size of each participant. The threshold for statistical significance was set to an uncorrected *p* < 0.001 at the initial voxel level and a false discovery rate-corrected p (FDR-p) < 0.05 (two-sided) at the cluster level.

Functional connectivity was assessed using mean resting-state time series extracted from supratentorial regions of interest (ROIs) from the Harvard-Oxford cortical and subcortical structural atlases^54–57^ Functional connectivity value was calculated by using Pearson’s coefficient of correlation followed by Fisher’s transformation^58^. To analyze functional connectivity between PD-p+ and PD-p−, analysis of covariance was performed with age and cognitive status (MMSE score) as covariates. The threshold for statistical significance was set to an FDR-p < 0.05 (two-sided).

Next, based on our hypothesis that altered attentional modulation to the FFA may cause pareidolia, we carried out ROI-to-voxel (seed-based) connectivity analysis using bilateral TFusC (including the FFA) as a seed. For the seed-based connectivity analysis between the PD-p+ and PD-p− groups, we performed an analysis of covariance using age and cognitive status (MMSE score) as covariates. The thresholds for statistical significance were set to an uncorrected *p* < 0.001 at the initial voxel level and an FDR-p < 0.05 (two-sided) at the cluster level.

To investigate the relationship between the severity of pareidolia and impaired functional connectivity, we performed Pearson’s correlation analysis between the pareidolia score and Z-score for functional connectivity of clusters detected in the seed-based analysis with the threshold for statistical significance set to *p* < 0.05. We also performed ANOVA for seed-based functional connectivity among the PD-p + , PD-p−, and HC groups, with post hoc Tukey–Kramer test. For multiple comparison, we used FDR adjustment with the statistical threshold of *p* < 0.05 (two-sided). We also calculated Cohen’s *d* as a measure of the effect size of the intergroup difference in functional connectivity. As our results showed left a dominant decrease in frontotemporal functional connectivity in the PD-p+ group, we performed ad hoc analysis comparing this connectivity between right- and left-dominant patients to investigate the effect of symptom laterality on the network alteration related with pareidolia.

In addition, since there were significant differences in cognitive status (MMSE scores) between the PD-p+ and PD-p− groups, we additionally conducted a post hoc analysis to distinguish the network associated with pareidolia from those associated with cognitive status. Using rsfMRI data, we performed a regression functional connectivity analysis for each pareidolia score and MMSE score. The threshold for statistical significance was set to an FDR-p < 0.05 (two-sided).

### Reporting Summary

Further information on research design is available in the Nature Research Reporting Summary linked to this article.

## Supplementary information

Reporting Summary

## Data Availability

The datasets used and/or analyzed during the current study are available from the corresponding author on reasonable request.
