# Supplementary material for: Decreased frontotemporal connectivity in patients with parkinson’s disease experiencing face pareidolia
Source: NPJ Parkinsons Dis. 2021 Oct 7;7:90. doi: 10.1038/s41531-021-00237-z (PMC8497472; doi:10.1038/s41531-021-00237-z)
Supplement: Supplementary file 1 — Reporting Summary [file 41531_2021_237_MOESM1_ESM.pdf]

nature research

Corresponding author(s): Masahito Mihara
Mideki Mochizuki

Last updated by author(s): Jul 2, 2021

Reporting Summary

Nature Research wishes to improve the reproducibility of the work that we publish. This form provides structure for consistency and transparency in reporting. For further information on Nature Research policies, see our Editorial Policies and the Editorial Policy Checklist.

Statistics

For all statistical analyses, confirm that the following items are present in the figure legend, table legend, main text, or Methods section.

n/a	Confirmed	
☐	☒	The exact sample size (n) for each experimental group/condition, given as a discrete number and unit of measurement
☒	☐	A statement on whether measurements were taken from distinct samples or whether the same sample was measured repeatedly
☐	☒	The statistical test(s) used AND whether they are one- or two-sided<br>*Only common tests should be described solely by name; describe more complex techniques in the Methods section.*
☐	☒	A description of all covariates tested
☐	☒	A description of any assumptions or corrections, such as tests of normality and adjustment for multiple comparisons
☐	☒	A full description of the statistical parameters including central tendency (e.g. means) or other basic estimates (e.g. regression coefficient) AND variation (e.g. standard deviation) or associated estimates of uncertainty (e.g. confidence intervals)
☐	☒	For null hypothesis testing, the test statistic (e.g. F, t, r) with confidence intervals, effect sizes, degrees of freedom and P value noted<br>*Give P values as exact values whenever suitable.*
☒	☐	For Bayesian analysis, information on the choice of priors and Markov chain Monte Carlo settings
☒	☐	For hierarchical and complex designs, identification of the appropriate level for tests and full reporting of outcomes
☐	☒	Estimates of effect sizes (e.g. Cohen's d, Pearson's r), indicating how they were calculated

Our web collection on statistics for biologists contains articles on many of the points above.

Software and code

Policy information about availability of computer code

Data collection	No software was used.
Data analysis	Matlab R2018a, Statistical Parametric Mapping 12, CONN-fMRI functional connectivity toolbox v17, R version 3.6

For manuscripts utilizing custom algorithms or software that are central to the research but not yet described in published literature, software must be made available to editors and reviewers. We strongly encourage code deposition in a community repository (e.g. GitHub). See the Nature Research guidelines for submitting code & software for further information.

Data

Policy information about availability of data

All manuscripts must include a data availability statement. This statement should provide the following information, where applicable:

- Accession codes, unique identifiers, or web links for publicly available datasets
- A list of figures that have associated raw data
- A description of any restrictions on data availability

The datasets used and/or analyzed during the current study are available from the corresponding author on reasonable request.

Field-specific reporting

Please select the one below that is the best fit for your research. If you are not sure, read the appropriate sections before making your selection.

☒ Life sciences ☐ Behavioural & social sciences ☐ Ecological, evolutionary & environmental sciences

For a reference copy of the document with all sections, see nature.com/documents/nr-reporting-summary-flat.pdf

Life sciences study design

All studies must disclose on these points even when the disclosure is negative.

Sample size	We recruited patients using consecutive cases enrolled in our cohort study during September 2014 and November 2016. Therefore, our study on 97 Parkinson's disease (PD) patients and 40 healthy subjects.
Data exclusions	We assessed quality check for MRI scans as follows; Participants with any artifacts in their MR scans or with excessive head movement creating more than 1 mm of displacement or 2.5° of rotation in any direction during resting-state functional MRI (rsfMRI) were excluded from further analysis. In this study, 97 patients with Parkinson's disease (PD) and 40 age- and sex-matched healthy individuals were recruited. Among the 97 selected patients with PD, 14 were excluded from further analysis due to poor quality of the imaging data. No participants in the healthy individual exhibited excessive head motion.
Replication	There was no cohort available with the same imaging protocol to replicate our results.
Randomization	No randomization was performed. This study is an observational study without any intervention.
Blinding	No blinding was performed. This study is an observational study without any intervention.

Reporting for specific materials, systems and methods

We require information from authors about some types of materials, experimental systems and methods used in many studies. Here, indicate whether each material, system or method listed is relevant to your study. If you are not sure if a list item applies to your research, read the appropriate section before selecting a response.

Materials & experimental systems

n/a	Involved in the study	
☒	☐	Antibodies
☒	☐	Eukaryotic cell lines
☒	☐	Palaeontology and archaeology
☒	☐	Animals and other organisms
☐	☒	Human research participants
☒	☐	Clinical data
☒	☐	Dual use research of concern

Methods

n/a	Involved in the study	
☒	☐	ChIP-seq
☒	☐	Flow cytometry
☐	☒	MRI-based neuroimaging

Human research participants

Policy information about studies involving human research participants

Population characteristics	This study was conducted as part of a longitudinal prospective cohort and registry study of PD at Osaka University that registered all patients with PD who were admitted to our hospital. From the patients registered between September 2014 and November 2016, we recruited those who complied with the following selection criteria: (1) age between 40 and 85 years, (2) diagnosed with PD in accordance with the United Kingdom Parkinson’s Disease Society Brain Bank criteria,41 (3) HY stage from 1 to 3, and (4) no clinical symptoms of dementia. Exclusion criteria were as follows: (1) a history of other neurological, psychiatric, or severe ocular disease; (2) the presence of subjective hallucinations based on interviews with the patient and caregiver; (3) current use of antipsychotic medications; and (4) a score of less than 24 on MMSE. According to these criteria, 97 patients with PD were selected from among 213 patients who were registered during this period. In addition, we selected 40 age- and sex-matched healthy individuals without any history of neurological or psychiatric diseases from another cohort study
Recruitment	We recruited all patients with PD admitted to our hospital who met the inclusion and exclusion criteria. Based on those criteria, our study included only patients without dementia or hallucinations. Therefore, there might be potential underestimation of cognitive dificit.
Ethics oversight	the Osaka University Hospital ethics review committee

Note that full information on the approval of the study protocol must also be provided in the manuscript.

Magnetic resonance imaging

Experimental design

Design type	Resting state
Design specifications	The experiment included one trial per subject. The length of a trial was 10 minutes and the number of the obtained volume was 240.
Behavioral performance measures	Not applicable

Acquisition

Imaging type(s)	functional and structural
Field strength	3 Tesla
Sequence & imaging parameters	Structural scans were obtained using a sagittal three-dimensional fast-spoiled gradient-recalled echo-pulse sequence with the following parameters: echo time (TE) = 2.7 ms, repetition time (TR) = 7.0 ms, inversion time = 400 ms, flip angle = 11°, matrix dimensions = 256 × 256, field of view (FOV) = 240 mm, slice thickness = 1.2 mm and slice number = 200. Functional imaging was obtained using axial gradient-echo echo-planar imaging under the following conditions: time points = 240, TE = 30 ms, TR = 2500 ms, flip angle = 80°, matrix = 64 × 64, FOV = 220 mm, slice thickness = 3.5 mm and slice number = 40.
Area of acquisition	A whole brain scan was used

Diffusion MRI ☐ Used ☒ Not used

Preprocessing

Preprocessing software	CONN-functional connectivity toolbox v17 software package was used with the "default preprocessing pipeline" for each preprocessing steps including brain extraction, segmentation and smoothing.
Normalization	Direct segmentation and normalization method was chosen in CONN-functional connectivity toolbox v17 software package. Functional and anatomical data are normalized into standard MNI space and segmented into grey matter, white matter, and CSF tissue classes using SPM12 unified segmentation and normalization procedure (Ashburner and Friston, 2005).
Normalization template	MNI space template provied by SPM12.
Noise and artifact removal	Linear regression of potential confounding effects in the BOLD signal and temporal band-pass filtering (0.009–0.08 Hz) in the default denoising pipeline of CONN-fMRI functional connectivity toolbox v17 software package was used for noise and artifact removal. Potential confounding effects in CONN's default denoising pipeline implement an anatomical component-based noise correction procedure (aCompCor) and include noise components from cerebral white matter and cerebrospinal areas (Behzadi et al. 2007), estimated subject-motion parameters (Friston et al. 1995) and identified outlier scans or scrubbing (Power et al. 2014).
Volume censoring	Participants with any artifacts in their MR scans or with excessive head movement creating more than 1 mm of displacement or 2.5° of rotation in any direction during rsfMRI were excluded from further analysis (n=14).<br>Censoring in CONN-fMRI functional connectivity toolbox v17 software package was used in the analysis, which is implemented by entering additional covariates (one covariate per outlier scan) in the Denoising regression step using Artifact Detection Tools to remove the influence of those outlier scans on the analyses. The additional covariates were 12 potential noise components defined from the estimated subject-motion parameters (3 translation, 3 rotation parameters and their associated first-order derivatives) and a variable number of noise components (one for each identified outlier scan during the outlier identification preprocessing step where acquisitions with framewise displacement above 0.9mm or global BOLD signal changes above 5 s.d. are flagged as potential outliers).

Statistical modeling & inference

Model type and settings	For structural MRI data, gray matter volume analysis was performed with a voxel-based morphometry technique using Statistical Parametric Mapping 12 (SPM12) as follows: segmentation into cerebrospinal fluid, gray matter, and white matter based on tissue probability maps; spatial normalization of gray matter segments to a gray matter template in the Montreal Neurological Institute space using diffeomorphic anatomical registration through exponentiated lie algebra techniques; and a 3D Gaussian filter of 8 mm full-width at half-maximum for smoothing of images.<br>For functional MRI data, we performed ROI-to-ROI based analysis and seed-based analysis in the first level analysis. ROI-to-ROI based analysis reveals the level of functional connectivity between each pair of ROIs, defined as the Fisher-transformed bivariate correlation coefficient between a pair of ROI BOLD timeseries. Seed-based analysis reveals the level of functional connectivity between a seed and every voxel in the brain by computing the Fisher-transformed bivariate correlation coefficients between an ROI BOLD timeseries and each individual voxel BOLD timeseries. The second-level random-effect analyses use the General Linear Model. All statistical modeling and analysis of functional MRI were conducted by using CONN-fMRI functional connectivity toolbox v17 software package.
Effect(s) tested	For both the ROI-to-ROI based and seed-based functional connectivity data, in the second level analysis, analysis of covariate

Effect(s) tested: was performed to compare PD patients with and without pareidolias with age and the Mini-Mental State Examination (MMSE) scores as covariates. In addition, simple regression analysis was performed between the ROI-to-ROI based functional connectivity data and the pareidolia scores or the MMSE scores.

Specify type of analysis: ☐ Whole brain ☐ ROI-based ☒ Both

Anatomical location(s): Supratentorial regions of interest from the Harvard-Oxford cortical and subcortical structural atlases

Statistic type for inference (See Eklund et al. 2016): Significance threshold set at voxelwise $p < 0.001$ (uncorrected), and threshold at clusterwise $p < 0.05$ (two-sided) with multiple comparisons by applying a false-discovery ratio (FDR) adjustment.

Correction: FDR correction for multiple comparisons for cluster-level statistics.

Models & analysis

n/a	Involved in the study
☐	☒ Functional and/or effective connectivity
☒	☐ Graph analysis
☒	☐ Multivariate modeling or predictive analysis

Functional and/or effective connectivity: Functional connectivity values were calculated by using Pearson's coefficient of correlation followed by Fisher's transformation.
